# Reduced Production of Pro-Inflammatory and Pro-Catabolic Factors by Human Serum Metabolites Derived from a Patented Saffron Extract Intake

**DOI:** 10.3390/pharmaceutics16030336

**Published:** 2024-02-28

**Authors:** Line Pourtau, Fabien Wauquier, Line Boutin-Wittrant, David Gaudout, Benjamin Moras, Adeline Vignault, Carole Vaysse, Tristan Richard, Arnaud Courtois, Stéphanie Krisa, Véronique Roux, Nicolas Macian, Gisèle Pickering, Yohann Wittrant

**Affiliations:** 1 Activ’Inside, 12 ZA Commerciale du Lapin, 33750 Beychac et Caillau, France; d.gaudout@activinside.com (D.G.); b.moras@activinside.com (B.M.); a.vignault@activinside.com (A.V.); 2 Clinic’n’Cell SAS, Faculty of Medicine and Pharmacy, TSA 50400, 28 Place Henri Dunant, 63001 Clermont-Ferrand, France; fabien.wauquier@clinicncell.com (F.W.); line.wittrant@clinicncell.com (L.B.-W.); yohann.wittrant@inrae.fr (Y.W.); 3 ITERG, Nutrition-Health & Lipid Biochemistry Department, 11 rue Gaspard Monge, ZA Pessac Canéjan, 33610 Canéjan, France; c.vaysse@iterg.com; 4 Bordeaux INP, INRAE, OENO, UMR 1366, ISVV, Bordeaux University, 33140 Villenave d’Ornon, France; tristan.richard@u-bordeaux.fr (T.R.); arnaud.courtois@u-bordeaux.fr (A.C.); stephanie.krisa@u-bordeaux.fr (S.K.); 5 Bordeaux Sciences Agro, 1 cours du Général de Gaulle, 33170 Gradignan, France; 6 CIC INSERM 1405, Plateforme d’Investigation Clinique CHU Gabriel Montpied, 58 Rue Montalembert, 63000 Clermont-Ferrand, France; v_morel@chu-clermontferrand.fr (V.R.); nmacian@chu-clermontferrand.fr (N.M.); gisele.pickering@uca.fr (G.P.); 7 INRAE, UMR 1019, UNH, 63009 Clermont-Ferrand, France; 8 Human Nutrition Unit, Clermont Auvergne University, BP 10448, 63000 Clermont-Ferrand, France

**Keywords:** clinical trial, inflammation, saffron extract, crocetin, safranal, joint, digestive, CaCo_2_, primary chondrocytes

## Abstract

Safe and anti-inflammatory plant-based natural products present an increasing focus in the treatment of chronic inflammatory diseases such as osteoarthritis or inflammatory bowel diseases. Among them, saffron, a spice derived from the stigma of *Crocus sativus,* could have anti-inflammatory properties and would be therefore a promising therapeutic agent for the treatment of such conditions. However, the anti-inflammatory molecular mechanisms of saffron in humans are still understudied and unclear. In this study, combining human serum metabolites and cell cultures, we evaluated the effect of circulating metabolites from the consumption of a patented saffron extract (Safr’Inside^TM^) on the chondrocytes and colon epithelial cell responses to inflammatory stress. Parametric or non-parametric Analysis of Variance with post hoc tests was performed. We demonstrated that human serum containing metabolites from saffron intake attenuated IL-1β-stimulated production of PGE2 and MMP-13 in chondrocyte cells and limited the increase in ICAM-1, MCP-1, iNOS, and MMP-3 in human epithelial cells following combined IL-1β and TNF-α inflammatory stimulation. Altogether, these data provide new findings into the mechanisms underlying the beneficial effects of saffron on chondrocytes and enterocyte cells at the cellular level and in the context of chronic inflammatory disorders.

## 1. Introduction

Inflammation is the immune system’s first response to harmful stimuli and therefore represents a defense mechanism that is vital to health [1]. However, when an initiating stimulus remains, or if the resolution process is disrupted, it can lead to chronic inflammation. Chronic inflammatory diseases have been rightly recognized as the most significant cause of death in the world today [2]. The characteristics of chronic inflammation include the on-site recruitment of primary inflammatory cells and the production of inflammatory cytokines or enzymes that contribute to the degradation of tissues [3]. Among chronic inflammatory diseases, we can cite immune-mediated inflammatory diseases including rheumatoid arthritis (RA) and inflammatory bowel disease (IBD) or cutaneous inflammatory abnormalities such as psoriasis and low-grade inflammatory-related diseases, including osteoarthritis or irritable bowel syndrome [4]. Although advances in the understanding of the molecular mechanisms of these pathologies have been made [5,6], most treatments proposed today aim only to reduce symptoms without specifically targeting the cause of the inflammation [7]. In addition, proposed pharmacotherapies can induce side effects that may be serious [8].

Interestingly, over the past few decades, several studies have investigated the relationship between lifestyle and the prevalence of chronic inflammatory diseases [9,10,11,12]. They have demonstrated that a healthy diet, daily exercise, or avoiding smoking can extend both an individual’s life span and lifetime in good health by preventing the incidence of chronic inflammatory diseases [13]. Among these modifiable determinants, nutrition may be the most influential [14]. Therefore, plant-based natural products with various anti-inflammatory effects and low toxicity have attracted increasing interest for the management and the support of treatment targeting chronic inflammatory diseases [15,16,17].

Among such natural products, saffron extract produced from the dried stigma of *Crocus sativus* L. seems to have some interesting properties and could be helpful in removing chronic inflammation triggers and reducing subsequent consequences [18]. Saffron stigma contains volatile compounds, mainly terpenes such as safranal, as well as non-volatile ones such as crocins, crocetins, picrocrocins, and flavonoids [18]. Interestingly, anti-arthritic and anti-inflammatory effects of crocin have already been demonstrated in an arthritis animal model. Hemshekhar et al. showed that treatment with crocin, after arthritis induction, neutralized the increased serum levels of different matrix metalloproteases such as MMP-13, MMP-3, and MMP-9 and pro-inflammatory cytokines [19]. The reduction in HO-1/Nrf-2 expression, as well as TNF-α, IL-6, and IL-1β inflammatory mediators, seems to be involved in the protective effect of crocetin [20]. In addition, safranal was shown to exert therapeutic effects in a rodent model of RA by reducing systemic levels of pro-inflammatory cytokines [21]. In a rodent model, saffron extract treatment decreased osteoarthritis-associated joint histological manifestations and decreased serum TNFα levels [22]. However, in humans, very few studies have been conducted. Recently, clinical outcomes and metabolic profiles in patients with active RA were evaluated after saffron supplementation. The authors concluded that saffron could positively alleviate the symptoms of patients, but the underlying mechanisms, especially in articular cartilage, were not elucidated [23]. Although saffron seems to be efficient, cellular mechanisms have never been evaluated, and therefore, they deserve to be investigated, in particular in osteoarthritis, since its prevalence in the population is higher than that of RA [24].

Remarkably, patients with RA have a higher risk of developing IBDs such as irritable bowel syndrome but also Crohn’s disease or ulcerative colitis [25]. Several studies have shown the efficacity of saffron compounds in various digestive inflammatory disorders. It has been shown that crocin may decrease a patient’s colitis disease activity index by eliciting anti-inflammatory responses in a dextran sulfate sodium-induced colitis mouse model [26]. In a randomized, double-blind, placebo-controlled study, patients suffering from mild-to-moderate ulcerative colitis were treated with saffron supplementation for 8 weeks. It was later shown that this improved the mean score of the simple clinical colitis activity index questionnaire, as well as leading to an improvement in antioxidant factors [27]. However, the effect of metabolized saffron extract in response to inflammatory stress remains largely uninvestigated in human epithelial cells.

To address both of these knowledge gaps and to contribute to the understanding of saffron’s mechanisms in humans, in particular in chronic inflammation diseases, we designed an ex vivo approach combining human serum compounds derived from the intake of a saffron extract and cell cultures and evaluated in this study whether these human circulating bioactives may influence the response to inflammatory stress in chondrocytes and intestine epithelial cells.

## 2. Materials and Methods

### 2.1. Study Design and Selection of the Ex Vivo Study

This study was conducted in accordance with the Declaration of Helsinki of 1975 (https://www.wma.net/what-we-do/medical-ethics/declaration-of-helsinki/, accessed on 1 April 2021), revised in 2013. The human study was approved by the French Ethical Committee (2021T2-02 RIPH2 HPS/N° SI RIPH: 21.01.11.58647/N° EudraCT/ID RCB: 2020-A3184-35/Comité de Protection des Personnes CPP Tours-Région Centre-Ouest I; approved 11 March 2021). Volunteers were informed of the objectives and the potential risks of the present study and provided their written informed consent before they participated in the study.

Relevance and robustness of such an ex vivo clinical trial were initially validated by Wittrant’s group [28,29,30,31,32,33]. A sample size of 10 subjects was hypothesized to detect a minimum effect size of 1, with a two-sided 5% significance level and a power of 90%. Healthy men (age: 25.0 years old, ±5.1; BMI: 23.9 kg/m^2^, ±2.3; >60 kg; without drug treatment; and no ethnic distinction) volunteered for this study. Their recruitment was validated based on normal full blood count, renal (urea and creatinine) and liver functions (aspartate aminotransferase (AST), alanine aminotransferase (ALT), and gamma-glutamyltransferase (GGT) activities). Volunteers fasted for 12 h before saffron extract intake. The patented saffron extract (SE), Safr’Inside^TM^ (patent WO2018020013), was provided by Activ’Inside (Beychac et Caillau, France). This hydroalcoholic extract is standardized to contain crocins (mainly trans-4-GG, trans-3-Gg; cis-4-GG, trans-2-G) > 3%, safranal > 0.2%, picrocrocin derivatives (mainly picrocrocin, HTCC) > 1%, and kaempferol derivatives (mainly kaempferol-3-sophoroside-7-glucoside, kaempferol-3-sophoroside) > 0.1%, measured by UHPLC method. Safr’Inside^TM^ was given at a dose of 300 mg (one capsule) and in one administration. The dose was set according to validated clinical data [23,27,34]. According to the crocetin’s absorption profile, determined during the first pharmacokinetic phase (on 10 volunteers) of the study, volunteers (n = 8) were subjected to a new blood sampling (venous blood from the cubital vein) before and after Safr’Inside^TM^ administration for the collection of both naïve (NHS) and maximum crocetin’s enriched serum fractions (EHS), respectively. Serum preparation and collection were performed at the Centre d’Investigation Clinique de Clermont-Ferrand—Inserm 1405—University hospital—France, to ensure the quality of samples and the compliance with regulatory and ethical laws (French standard certification NF-S-96900). Serum was stored at −80 °C until ready for analysis.

### 2.2. Cell Culture and Treatment

#### 2.2.1. Human Chondrocytes

Human articular chondrocytes (HACs) were harvested from tibial plateau and femoral condyles following a knee replacement surgery (University hospital, Clermont-Ferrand, France) and isolated as previously described [35]. Only intact cartilage areas were kept and processed for chondrocyte isolation. Briefly, cartilage was sliced, and chips were successively digested at 37 °C with 0.05% type IV-S hyaluronidase (750–3000 units/mg) (Sigma-Aldrich, Lyon, France) in Hank’s Balanced sodium Salt (HBSS) (Life Technologies, Villebon-Sur-Yvette, France) for 10 min, with 0.2% trypsin (≥9000 BAEE units/mg) (Sigma-Aldrich, Lyon, France) for 15 min and with 0.2% type II collagenase (125 units/mg) (Sigma-Aldrich, Lyon, France) for 30 min. Cartilage chips were then digested overnight at 37 °C in 0.03% type II collagenase in control medium (DMEM) (Life Technologies, Villebon-Sur-Yvette, France) supplemented with 10% fetal calf serum (FCS) (Pan-Biotech, Aidenbach, Germany) and 1% penicillin/streptomycin (P/S) (Life Technologies, Villebon-Sur-Yvette, France). Cells were plated at passage 1 in F225 flasks at a density of 100,000 cells/cm^2^ and maintained at 37 °C in a humidified atmosphere of 5% CO_2_ in a control medium (10% FCS, 1% P/S). At confluency, cells were subcultured for ex vivo experiments.

**Figure 1:**
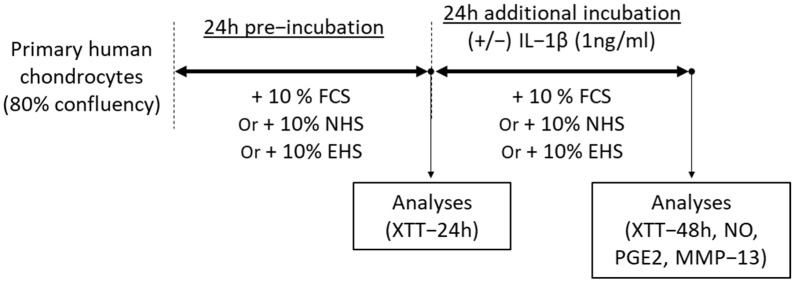
Time line of experiment for chondrocyte investigations. Human articular chondrocytes were seeded for ex vivo experiments in culture medium with 10% of FCS and grown until they reached 80% confluence. Cells were then incubated for 24 h in culture medium in the presence of 10% of FCS, NHS, or EHS before analyses of viability (XTT-24 h). Cells were then incubated for an additional 24 h, still in the presence of 10% of FCS, NHS, or EHS (culture medium being changed) but with or without IL-1β (1 ng/mL). Analyses of viability (XTT-48 h), NO, PGE2, and MMP-13 were carried out at the end of this new incubation period. FCS: fetal calf serum, NHS: naïve human serum, EHS: enriched human serum, IL-1β: Interleukin-1 β, NO: nitric oxide: PGE2: Prostaglandin E2, MMP-13: Metalloprotease-13.

To validate the inflammatory model, the IL-1β induction was first tested in DMEM media containing 10% FCS [31]. Upon validation, in order to be able to analyze the effects of saffron extract’s metabolites, cells were preincubated for 24 h in DMEM in the presence of 10% of human serum (naïve or enriched) according to the Clinic’n’Cell protocol (DIRV INRA 18-00058) and 1% P/S prior to an additional 24 h treatment with human recombinant IL-1β (reference IL038, Millipore Corporation, Molsheim, France) at 1 ng/mL (Figure 1). For each condition tested on chondrocytes, biological replicates were prepared with different cell culture wells. For FCS conditions, measurements described below were performed with 4 biological replicates. For human serum conditions, measurements described below were performed with 4 biological replicates for each volunteer (n = 8 volunteers). The volunteers’ serums were not pooled.

#### 2.2.2. Human Enterocytes

Caco-2 are epithelial cells that were isolated from colon tissue derived from a 72-year-old, white male with colorectal adenocarcinoma [36]. They were obtained from the ATCC (reference HTB-37™, American Type Culture Collection, Manassas, VA, USA) and were plated at passage 6 in F225 flasks at a density of 100,000 cells/cm^2^ and maintained at 37 °C in a humidified atmosphere of 5% CO_2_ in control medium (10% FCS, 1% P/S).

**Figure 2:**
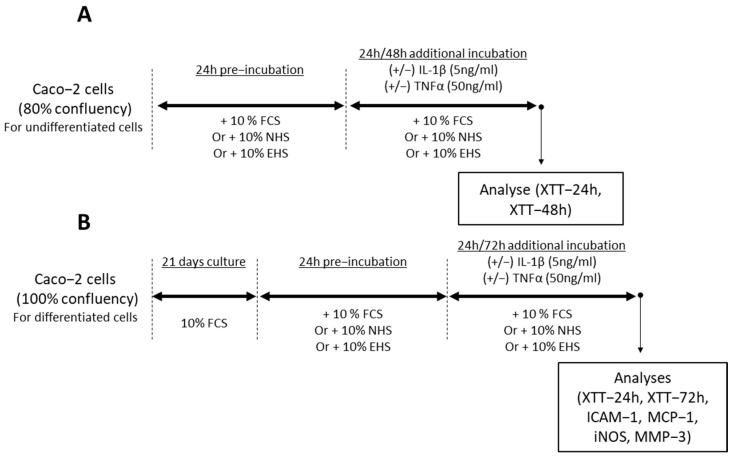
Time line of experiment for enterocyte investigations. (**A**) Undifferentiated enterocytes. (**B**) Differentiated enterocytes. For the undifferentiated enterocytes, cells were first incubated for 24 h in culture medium in the presence of 10% of FCS, NHS, or EHS, followed by a new incubation period of 24 h or 48 h with or without IL-1β (5 ng/mL)/TNFα (50 ng/mL), still in the presence of 10% of FCS, or NHS, or EHS. Analyses of viability (XTT-24 h, XTT-48 h) were performed at the end of this new incubation period. For the differentiated enterocytes, this was carried out after confluency cells were left to grow for 21 days in the presence of FCS (10%). Then, as for undifferentiated enterocytes, cells were incubated for 24 h in culture medium in the presence of 10% of FCS, NHS, or EHS, followed by a new incubation period of 24 h or 72 h with or without IL-1β (5 ng/mL)/TNFα (50 ng/mL) before analyzing the viability (XTT-24 h, XTT-72 h), ICAM-1, MCP-1, iNOS, and MMP-3. FCS: fetal calf serum, NHS: naïve human serum, EHS: enriched human serum, IL-1β: Interleukin-1 β, TNFα: Tumor necrosis factor α, ICAM-1: intercellular adhesion molecule-1, MCP-1: monocyte chemoattractant protein-1, iNOS: inducible nitric oxide synthase, MMP-3: matrix metalloproteinase-3.

To validate the inflammatory model, the induction, achieved by the combination of human recombinant TNF-α 50 ng/mL (reference ab259410, Abcam, Paris, France) and human recombinant IL-1β 5 ng/mL (reference IL038, Millipore Corporation, Molsheim, France), was first tested in DMEM media containing 10% FCS [31,37]. Upon validation, in order to analyze the effects of saffron extract’s metabolites on undifferentiated enterocytes, at 80% of confluency, cells were preincubated for 24 h in DMEM in the presence of 10% of human serum (naïve or enriched) and 1% P/S and then treated with a mix of TNF-α 50 ng/mL and IL-1β 5 ng/mL for an additional period of either 24 or 48 h (Figure 2A).

To analyze the effects of saffron extract’s metabolites on more established enterocytes, cells were grown to reach confluency and then cultured for 21 days in DMEM (15% FCS; 1% P/S). At the end of the differentiation period, cells were preincubated for 24 h in DMEM in the presence of either 10% FCS or 10% of human serum (naïve or enriched) (1% P/S) and treated with a mix of TNF-α 50 ng/mL and IL-1β 5 ng/mL for an additional 24 or 72 h period (Figure 2B). For each condition that was tested on enterocytes, biological replicates were prepared with different culture wells. For FCS conditions, measurements described below were performed with 4 biological replicates. For human serum conditions, measurements described below were performed with 4 biological replicates for each volunteer (n = 8 volunteers). The volunteers’ serums were not pooled.

### 2.3. Measurement of Cell Viability

Cell viability was investigated based on an XTT-based method (Cell Proliferation Kit II, Sigma-Aldrich, Lyon, France), and experimental procedures were set up according to the supplier’s recommendations [31]. Optical density was measured at 450 nm by spectrophotometer ELX808 using KC Junior Software (V1.41.8) (Biotek Instruments Inc., Winooski, VT, USA).

### 2.4. Measurement of Nitric Oxide (NO) and Prostaglandin E2 (PGE2)

Nitrate/Nitrite (final products of NO) colorimetric assay [38] and prostaglandin E2 Enzyme Immunoassay (EIA) kits [39] were obtained from Cayman Chemical (references 780001 and 514010 respectively, Cayman Chemical, Ann Arbor, MI, USA). Measurements in cell supernatants were performed according to manufacturer’s instructions using a spectrophotometer ELX808 and running KC Junior Software (V1.41.8) (Biotek Instruments Inc., Winooski, VT, USA).

### 2.5. Measurement of Intercellular Adhesion Molecule-1 (ICAM-1), Monocyte Chemoattractant Protein-1 (MCP-1), and Matrix Metalloproteinases (MMP-13)

Human ELISA Kits for ICAM-1 [40], MCP-1 [41], and MMP-13 [42] detection were purchased from Abcam (references ab174445, ab179886, and ab221839, respectively, Abcam, Paris, France). Measurements in cell supernatants were performed according to manufacturer’s instructions using a spectrophotometer ELX808 and running KC Junior Software (V1.41.8) (Biotek Instruments Inc., Winooski, VT, USA).

### 2.6. Real Time RT-PCR

mRNA either from chondrocytes or enterocytes were isolated using TRIZOL (Invitrogen, Ilkirsh, France) according to supplier’s recommendations. Inducible nitric oxide synthase (iNOS) and matrix metalloproteinase-3 (MMP-3) mRNA expression levels were measured by RT-qPCR. RNA was reverse-transcribed into complementary DNA using maxima first-strand cDNA synthesis kit reaction mix [43] (ThermoScientific, Illkirch, France) according to supplier’s recommendations. Gene amplification were performed using PowerUp SYBRgreen master mix (Applied Biosystems, Waltham, MA, USA) according to supplier’s recommendations on a QuantStudio3 device running Design and Analysis 2 (DA2) software (V2.5.1) (Applied Biosystems, Illkirch, France). Β-Actine was used as a housekeeping gene. Primers (Integrated Dna Technologies, Leuven, Belgium) were designed as follows: iNOS-F: 3′-TCT CAA GGC ACA GGT CTC TTC-5′; iNOS-R: 3′-GTT CTT CAC TGT GGG GCT TG-5′; MMP-3-F: 3′-TGA GTG AGT GAT AGA GTG GGT-5′; MMP-3-R: 3′-TGA ACA ATG GAC AAA GGA TAC AAC-5′; ACTβ-F: 3′-ATT GGC AAT GAG CGG TTC-5′; and ACTβ-R: 3′-GGA TGC CAC AGG ACT CCA5′.

### 2.7. Statistics

Prism V.9.4.1 (GraphPad Software, Boston, MA, USA) was used to run statistical tests and draw figures. The following statistical plan was applied: A Shapiro–Wilk normality test was used to determine whether the data were consistent with a Gaussian distribution. When normal distribution and equal variance were assumed, measures were subjected to unpaired t-test or one-way ANOVA with Tukey’s test for multiple comparisons. If data were not distributed according to a normal distribution, a Mann–Whitney or Kruskal–Wallis non-parametric test, followed by Dunn test for post hoc for multiple comparisons, were used. Values in the text are presented as the means unless specified otherwise, and results in the figures are presented as box and whisker plots, where whiskers are min to max to visualize the variability in groups. Differences were considered statistically significant at *p* ≤ 0.05, with * for *p* < 0.05; ** for *p* < 0.01; *** for *p* < 0.001; **** for *p* < 0.0001; and non-significant (ns) for *p* > 0.05.

## 3. Results

### 3.1. Validation of Ex Vivo Procedures with Human Sera on Chondrocyte and Enterocyte Cells

**Figure 3:**
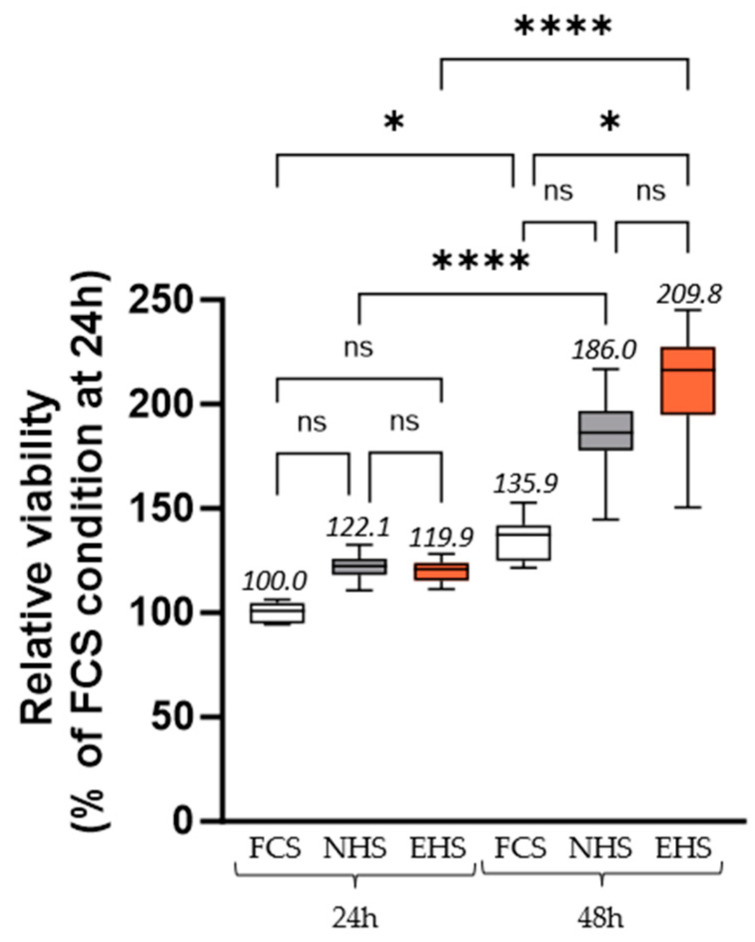
Relative chondrocyte viability measured by XTT-based assay after 24 h and 48 h of serum treatment. Twenty-four-hour incubation with FCS 10% was set as the control condition, and results of other conditions are expressed in relation to this control condition. Values are presented as the box and whisker plots, with min to max and group means above. Differences were considered statistically significant at *p* < 0.05 with * for *p* < 0.05, **** for *p* < 0.0001 and ns for *p* > 0.05. FCS: fetal calf serum (in white), NHS: naïve human serum (in grey), EHS: enriched human serum (in orange).

To ensure further investigations regarding the clinical ex vivo approach, we first validated that the human sera did not negatively affect cell growth compared to regular FCS incubation. Neither NHS nor EHS exerted any adverse effects on the chondrocyte cells’ growth at 24 h and 48 h (Figure 3). At 48 h, the presence of EHS increased the cell growth compared to FCS by 54.4%. At the same time, a slight EHS-induced promotion of cell growth was also observed compared to NHS (+12.8%), although it was not significant. A previous experiment showed that pro-inflammatory stress induced by the addition of IL-1β at 1 ng/mL did not affect the viability of chondrocyte cells [44].

**Figure 4:**
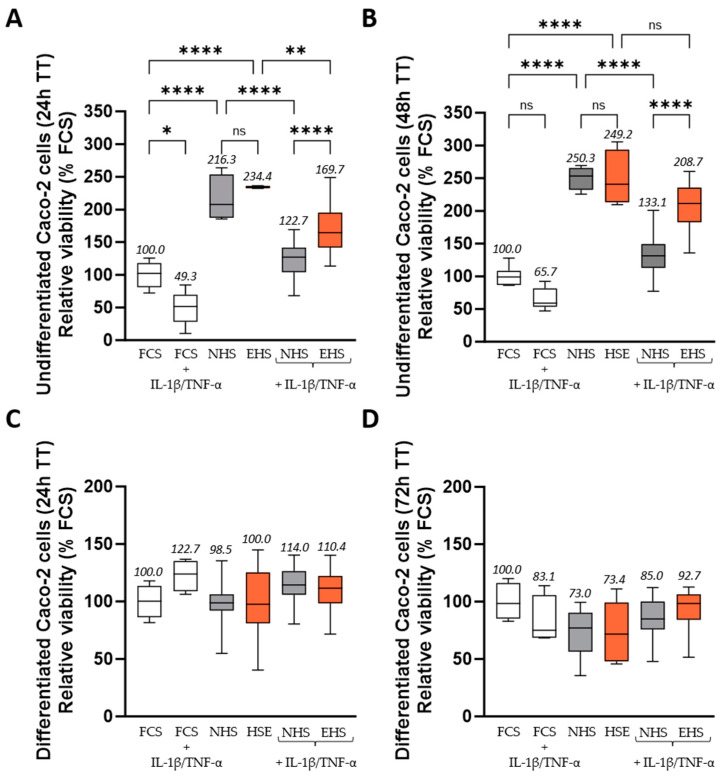
Relative enterocyte viability measured by XTT-based assay at 24 h (**A**,**C**), 48 h (**B**), and 72 h (**D**), with or without inflammatory treatment. Viability was investigated either at 80% confluency (undifferentiated enterocytes (**A**,**B**)) or after a culture of 21 days post-confluency (differentiated enterocytes (**C**,**D**)). Inflammatory stress was induced by the addition of both human recombinant TNF-α (50 ng/mL) and human recombinant IL-1β (5 ng/mL). Incubation with FCS 10% in the absence of inflammatory stimulation was set as the control condition, and the results of other conditions were expressed in relation to this control condition. Values are presented as box and whisker plots, with min to max and group means above. Differences were considered statistically significant at *p* < 0.05, with * for *p* < 0.05; ** for *p* < 0.01; **** for *p* < 0.0001; and ns for *p* > 0.05. FCS: fetal calf serum (in white), NHS: naïve human serum (in grey), EHS: enriched human serum (in orange).

Along with chondrocyte cultures, we also confirmed that neither NHS nor EHS negatively impacted undifferentiated or differentiated enterocyte cultures. In their undifferentiated state, the Caco-2 cell growth was more than doubled in the presence of NHS compared to FCS (both at 24 h (+116%) (Figure 4A) and 48 h (+150%) (Figure 4B)). The pro-inflammatory stress induced by the addition of both TNF-α 50 ng/mL and IL-1β 5 ng/mL strongly impaired cell growth; 50.7% less in the presence of FCS; and 43.3% less in the presence of NHS at 24 h. At 48 h post pro-inflammatory treatment, the cell growth remained reduced compared to the non-inflammatory conditions. Reductions in cell growth by 34.3% in the presence of FCS and by 46.8% in the presence of NHS were observed. The presence of saffron extract metabolites had no influence on the cell growth in the absence of stress (24 and 48 h) compared to the NHS condition. In contrast, it potently prevented the TNF-α/IL-1β effect and contributed to restoring cell growth by limiting the decrease to −27.6% at 24 h and −16.2% at 48 h (halfway to the control condition) (Figure 4A,B). When cells were cultured for 3 weeks to maintain them in a more differentiated state, conditions were no longer significantly different from each other (Figure 4C,D).

### 3.2. Effect of Human Serum Containing Compounds from Saffron Extract Ingestion on Inflammation Response in Chondrocytes

**Figure 5:**
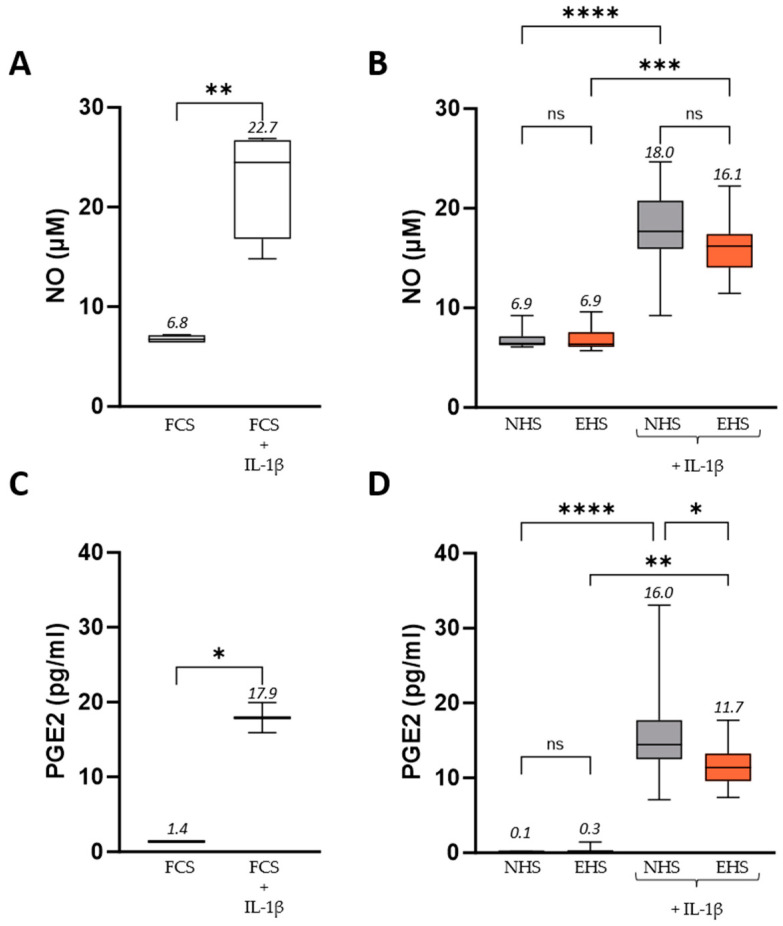
Exploration of secondary inflammatory markers in human primary chondrocytes (nitric oxide: NO (**A**,**B**) and Prostaglandin E2: PGE2 (**C**,**D**)). Incubation with FCS 10% served as control conditions for the validation of both the inflammatory model and the human serum incubation. Inflammatory stress was induced by the addition of human recombinant IL-1β (1 ng/mL). Values are presented as box and whisker plots, with min to max and group means above. Differences were considered statistically significant at *p* < 0.05, with * for *p* < 0.05; ** for *p* < 0.01; *** for *p* < 0.001; **** for *p* < 0.0001; and ns for *p* > 0.05. FCS: fetal calf serum (in white), NHS: naïve human serum (in grey), EHS: enriched human serum (in orange).

As described in the literature and acknowledged in clinic [45,46,47], IL-1β promotes the production of both PGE2 and NO by chondrocytes. As expected, upon IL-1β stimulation, NO increased from 6.8 µM to 22.7 µM when cells were incubated with FCS (Figure 5A) and from 6.9 µM to 18 µM in the presence of NHS (Figure 5B). Moreover, IL-1β stimulation induced an increase in PGE2 from 1.4 pg/mL to 17.9 pg/mL in the presence of FCS (Figure 5C) and from 0.1 pg/mL to 16.0 pg/mL when cells were incubated with NHS (Figure 5D). These results validated our ex vivo model. The presence of circulating bioactives from saffron significantly countered the rise in PGE2. Indeed, the level of PGE2 was 11.7 pg/mL in the presence of EHS, while it was 16.0 pg/mL when the cells were incubated with NHS, representing a reduction of 26.9%. Without IL-1β stimulation, no significant effects of circulating bioactives from saffron on NO and PGE2 production were observed.

**Figure 6:**
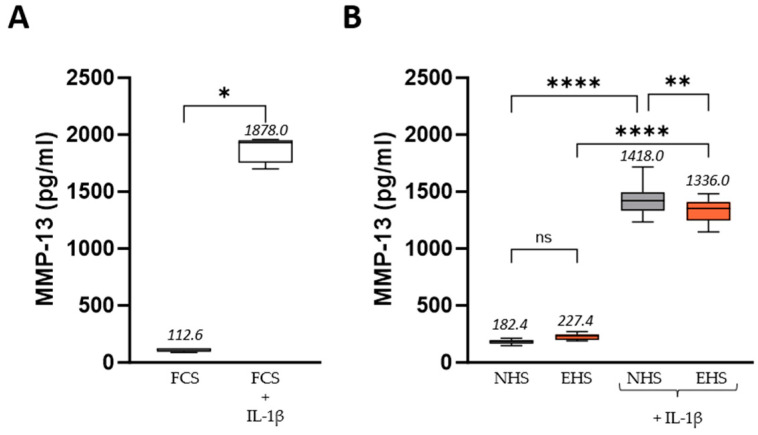
MMP-13 protein released by human primary chondrocytes upon FCS (**A**) or human serum incubation (**B**). Incubation with FCS 10% served as control conditions for the validation of both the inflammatory model and the human serum incubation. Inflammatory stress was induced by the addition of recombinant human IL-1β (1 ng/mL). Values are presented as box and whisker plots, with min to max and group means above. Differences were considered statistically significant at *p* < 0.05, with * for *p* < 0.05; ** for *p* < 0.01; **** for *p* < 0.0001; and ns for *p* > 0.05. FCS: fetal calf serum (in white), NHS: naïve human serum (in grey), EHS: enriched human serum (in orange).

IL-1β stimulation also drives the increase in catabolic factors that has already been described for their role in cartilage breakdown in osteoarthritis. In this study, the MMP-13 protein level significantly increased from 112.6 pg/mL to 1878.0 pg/mL upon IL-1β stimulation in the FCS condition (Figure 6A), while a similar upregulation was observed with NHS (182.4 vs. 1418.0 pg/mL) (Figure 6B). As for PGE2, the presence of EHS slightly but significantly limited the IL-1β-related effects (1418.0 vs. 1336.0 pg/mL), with a reduction in the MMP-13 level by 5.8 % compared to NHS alone. In the absence of IL-1β stimulation, no significant effect of the circulating bioactives from saffron on MMP-13 production was observed.

### 3.3. Effect of Human Serum Containing Compounds from Saffron Extract Ingestion on Expression and Production of Pro-Inflammatory and Catabolic Mediators Induced by Inflammation Treatment on Differentiated Enterocyte Cells

**Figure 7:**
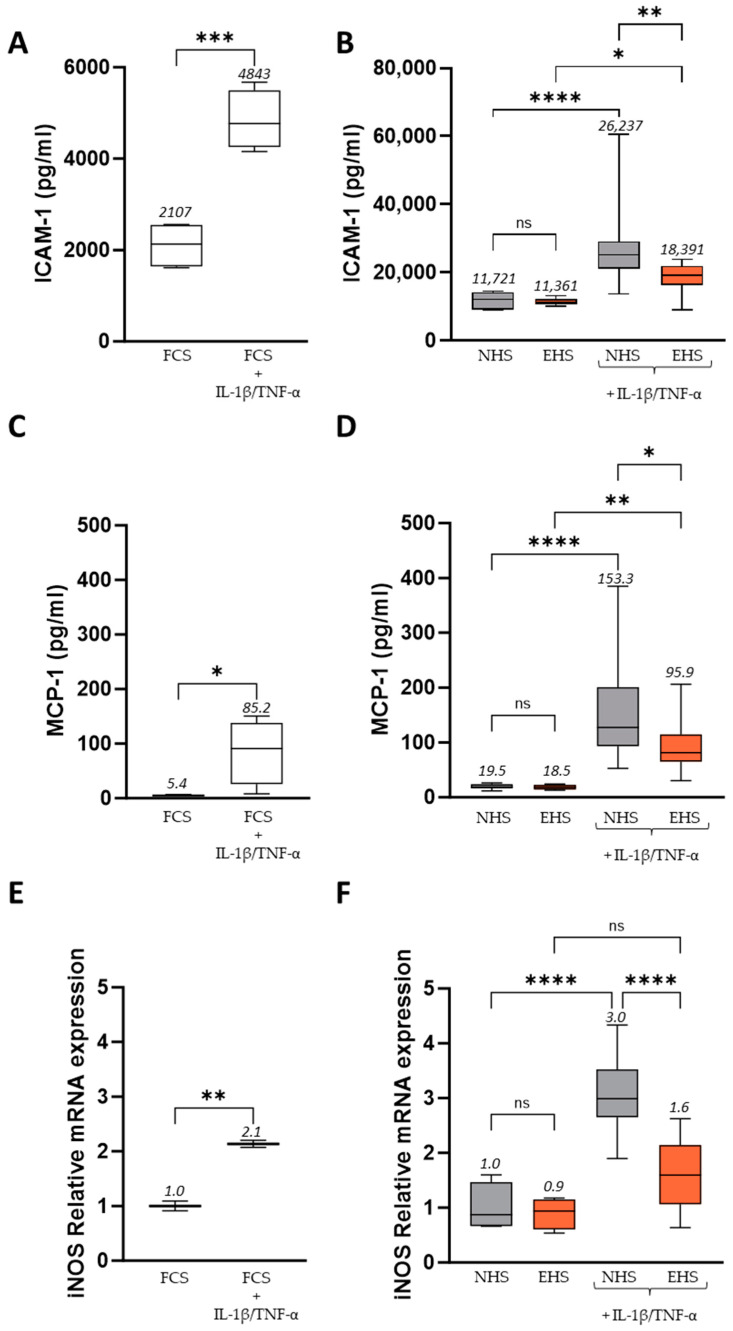
Exploration of inflammatory markers in human enterocytes upon FCS (**A**,**C**,**E**) or human serum incubation (**B**,**D**,**F**). ICAM-1 (**A**,**B**) and MCP-1 (**C**,**D**) protein levels were evaluated in cell supernatants. iNOS mRNA levels were evaluated by RT-PCR (**E**,**F**). Inflammatory stress was induced by the addition of both recombinant human TNF-α (50 ng/mL) and recombinant human IL-1β (5 ng/mL). Incubation with FCS 10% served as control conditions for the validation of both the inflammatory model and the human serum incubation. Values are presented as box and whisker plots, with min to max and group means above. Differences were considered statistically significant at *p* < 0.05, with * for *p* < 0.05; ** for *p* < 0.01; *** for *p* < 0.001; **** for *p* < 0.0001; and ns for *p* > 0.05. FCS: fetal calf serum (in white), NHS: naïve human serum (in grey), EHS: enriched human serum (in orange).

ICAM-1, MCP-1, and iNOS are well-known inflammatory targets in IBD. In differentiated enterocyte cultures, the presence of TNF-α + IL-1β enhanced ICAM-1 production (+130% in FCS condition; +124% in the presence of NHS) (Figure 7A,B). EHS significantly alleviated this rise. Indeed, ICAM-1 production was reduced by 29.9% when the cells were incubated with human serum containing saffron metabolites compared to the NHS condition (26,237 vs. 18,391 pg/mL). Thus, the rise in ICAM-1 in inflammatory conditions was halved in the presence of serum enrichment with saffron bioactives.

The same observation was made for MCP-1 release. TNF-α + IL-1β induced a strong increase in MCP-1 in the FCS and NHS conditions, which was significantly constrained by SE metabolites (Figure 7C,D). MCP-1 release was reduced by 37.4% when the cells were incubated with human serum containing metabolites from saffron compared to the NHS condition (153.3 vs. 95.9 pg/mL).

As expected, the inflammatory stress also led to a potent and significant increase in iNOS mRNA expression (2.1 times more expressed in FCS condition and 3 times more expressed in NHS condition) (Figure 7E,F). Upon TNF-α/IL-1β incubation, we found that the increase in iNOS expression was significantly reduced in the presence of EHS (by 46.7%) compared to NHS. The absence of a significant difference between human serum containing saffron metabolites without inflammation and this same condition under TNF-α/IL-1β stimulation suggests that saffron metabolites completely protect against the elevation of these markers in inflammatory conditions.

**Figure 8:**
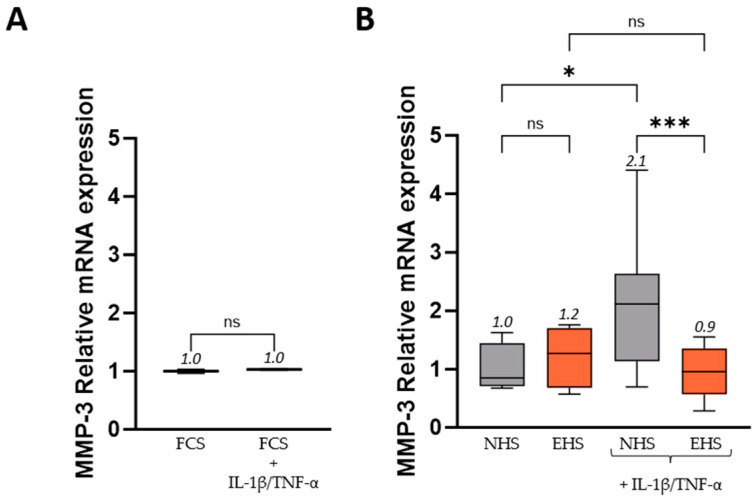
MMP-3 relative mRNA expression in human enterocytes following FCS (**A**) or human serum incubation (**B**). Inflammatory stress was induced by the addition of both recombinant human TNF-α (50 ng/mL) and recombinant human IL-1β (5 ng/mL). Values are presented as box and whisker plots, with min to max and group means above. Differences were considered statistically significant at *p* < 0.05, with * for *p* < 0.05; *** for *p* < 0.001; and ns for *p* > 0.05. FCS: fetal calf serum (in white), NHS: naïve human serum (in grey), EHS: enriched human serum (in orange).

In inflamed tissues of IBD patients, MMPs are produced in excess and make a major contribution to the IBD-related mucosal degradation. Consistent with the literature, the cocktail of TNF-α + IL-1β increased MMP-3 expression by +110% in the presence of NHS [48,49]. This increase was completely clear when cells were cultured in the presence of SE human bioactives, returning the MMP-3 expression to its baseline (Figure 8).

In the absence of inflammatory stress, human metabolites from saffron intake had no effect compared to naïve serum, regardless of the marker considered (ICAM-1, MCP-1, iNOS, MMP-3).

## 4. Discussion

Although nutrition may influence the incidence of chronic inflammatory diseases, the effects of plant-based natural products remain poorly understood at the cellular level and especially in clinical settings. This study addressed the in vitro induction of NO, PGE2, and MMP-13 in human chondrocyte cells by IL-1β and demonstrated that human serum containing compounds from Safr’Inside^TM^ intake attenuated IL-1β-stimulated production of PGE2 and MMP-13. We also demonstrated that those human circulating compounds limit the increase in ICAM-1, MCP-1, iNOS, and MMP-3 in enterocyte cells following a combined IL-1β and TNF-α stimulation.

It is well established that MMPs, especially MMP-13, also known as collagenase-3, play an important role in the progression of cartilage degradation in RA or osteoarthritis [50,51]. MMP-13 is considered the most important mediator in the pathogenesis of these diseases [52,53]. Indeed, treatment of RA mice with selective inhibitors of MMP-13 reduced the mean arthritic score in comparison to control mice [54], and MMP-13 knockout mice were protected from collagen antibody-induced arthritis [55], suggesting that MMP-13 inhibition could have a therapeutic value. In the present study, treatment of human chondrocyte cells with human serum containing compounds from Safr’Inside ^TM^ intake reduced the production of MMP-13 in inflammatory stress conditions. Reduced MMP-13 production and expression following IL-1β stimulation have been already observed on chondrocytes from rabbit after incubation with crocin [56]. However, animal studies conducted on the bioavailability of saffron compounds showed that after oral administration of crocins or crocetins, only crocetins were found in the plasma. Orally administered crocins are hydrolyzed to crocetins before or during intestinal absorption, and absorbed crocetins are partly metabolized to mono- and diglucuronides [57]. The effect of treatment with crocin, directly on chondrocytes, is therefore not translatable to in vivo systems, since crocin, per os, does not reach the bloodstream. Here, due to the original approach, using serum from volunteers receiving Safr’Inside ^TM^, the effect of all circulating saffron-derived metabolites was evaluated.

Regarding the potential mechanisms of saffron-derived metabolites that are involved in the regulation of the MMP-13 increase after stimulation of IL-1β, the regulation of the nuclear factor-kappaB (NK-κB) signaling pathway could represent a probable target. Indeed, studies have identified NF-κB as abnormally activated in osteoarthritis [58,59], and several studies have demonstrated that the increase in MMP-13 expression after IL-1β was dependent of the activation of this transcription factor [51]. Interestingly, Li et al. showed that arthritic rats treated with crocetin had a decreased expression of NF-κB compared to untreated arthritic rats [20]. Therefore, although unexplored in this study, saffron extract could act on human chondrocytes in the same way.

The NF-κB pathway is also essential for the chondrocytes to express other inflammation-related genes, such as iNOS and COX2. It is well described that osteoarthritis chondrocytes overexpress iNOS [60] and its product, NO, which may activate matrix metalloproteases [61]. In the present study, although not significant, human serum containing compounds from saffron extract intake seemed to attenuate the NO production, suggesting that these metabolites could reduce catabolic processes that are triggered by inflammatory stimuli. COX2 leads to the synthesis of PGE2, which, by binding to the EP2 and EP4 PGE2 receptors that are located in the joint tissues, also participate in cartilage degradation and osteoarthritis progression. In line with our results, in stimulated cells culture of macrophages, a treatment with crocetins reduced COX2, PGE2, iNOS, and NO production [20].

In addition, some studies reported that the silent information regulator factor 2-related enzyme 1 (Sirt1), which belongs to the family of NAD^+^-dependent class 3, had a positive role in osteoarthritis by maintaining chondrocyte homeostasis [62,63]. Indeed, Sirt1 could activate deacetylation of non-histone proteins, such as p53 or NF-κB [64,65]. Interestingly, safranal could upregulate Sirt1 expression in chondrocytes, thus preventing chondrocyte apoptosis and extracellular matrix degeneration [66]. Safranal is a compound that is present in the extract and may participate in the observed effects on chondrocytes.

In immune-mediated inflammatory diseases, patients often present concomitant pathologies. Thus, patients with RA have a higher risk of developing irritable bowel syndrome and other IBDs. For this reason, we also explored the effect of human serum containing saffron compounds on epithelial cells that are responsible for absorptive processes and the production of mucus in inflammatory conditions.

The signaling events that are induced by inflammatory mediators, in an osteoarthritis context, lead to the NF-κB activation and to pro-inflammatory gene expression as previously mentioned, but this is also the case in IBD [67]. In turn, NF-κB regulates the expression of ICAM-1, a cell surface glycoprotein that plays a pivotal role in the recruitment of leucocytes at the sites of intestinal inflammation [68]. Studies in a mouse model of dextran sulphate sodium-induced colitis demonstrated increased ICAM-1 expression with disease activity [69]. In humans, ICAM-1 has been found to increase in colonic lysates from ulcerative colitis patients compared to non-inflammatory controls [70]. Together, this evidence suggests that ICAM-1 inhibition could be an attractive therapeutic option for IBDs. In other models, a few studies have demonstrated that saffron compounds could decrease ICAM-1 expression [71,72]. However, to the best of our knowledge, we have demonstrated for the first time the positive effect of human serum containing compounds from saffron extract intake on ICAM-1 markers in inflamed human epithelial cells, which could help in the treatment of IBDs. We also found in our cell model a limited increase in iNOS in the presence of saffron’s human circulating metabolites, supporting the hypothesis that saffron metabolites could act on the NF-κB signaling pathway in human cells.

MCP-1 expression has been reported to be increased in IBDs as well [73]. Stimuli for epithelial cell chemokine production include IL-1β and TNF-α, and as expected, we observed an increase in MCP-1 production in CaCo_2_-stimulated cells, which was attenuated in the presence of human serum containing metabolites. Here again, very few studies have already been conducted on the potential effects of saffron on MCP-1 production [74,75,76,77], but none have investigated saffron’s effect on enterocytes.

Studies that are focused on the effects of saffron or its compounds on inflammatory bowel disorders show that Nrf2, one of the pivotal transcription factors regulating the expression of antioxidant proteins that are triggered by injury and inflammation, could be involved in the beneficial effects of crocin. Indeed, in an ulcerative colitis model, Khodir et al. observed that crocin significantly restored the colon’s Nrf2 content and enhanced HO-1 activity, thus inhibiting the production of pro-inflammatory mediators [78]. An increased Nrf2 expression in the colorectal mucosa of mice along with a decreased expression of TNF-α, IL-1β, IL-6, IFN *γ*, NF-κB, COX2, and iNOS were also found after a 4 week supplementation with crocin in DSS-induced colitis [79]. The data reported in this ex vivo clinical study are consistent with published preclinical results, but further investigations are needed to support the mechanisms of action, especially on transcription factors.

To go further into the potential saffron mechanisms involved in chronic inflammatory diseases, highlighting the role of microbiota in these pathologies seems crucial. Indeed, a growing body of evidence indicates that the gut microbiota plays an important role, not only in gastrointestinal diseases including irritable bowel syndrome [80,81] but also in joint diseases such as osteoarthritis [82]. Recent evidence shows that patients with an IBD have a reduced level of *Akkermensia. muciniphila,* suggesting that these bacteria may contribute to the process and development of IBDs [83]. Moreover, modifications of microbiota observed in patients with RA also included a reduction in the genera *Akkermansia* [82]. Crocin seems to inhibit DSS-induced colitis by directly affecting the absorption of DSS, and these modifications could be possible due to alterations in the intestinal microflora [79]. Interestingly, we have recently explored the effect of saffron extract supplementation on microbiota in a rodent model of low-grade chronic inflammation and have demonstrated that in saffron extract-treated mice, there was an increase in *Akkermansia* compared to in the LPS-treated group [84]. Therefore, in addition to the direct effect of saffron’s metabolites both on chondrocytes and enterocytes, saffron could help patients with chronic inflammatory diseases by maintaining in part a normal intestinal microflora. Moreover, other recent studies have also focused on the immunomodulatory properties of products that have been isolated from probiotics, termed postbiotics [85]. These compounds, such as lysates, could have significant influence on inflammation levels [86], making them interesting compounds as adjuvants, also in combination with saffron extract, in future trials.

Although we have shown beneficial effects on cells in this study, one of the main limitations of this study is that we are unable to identify the specific compound(s) that are responsible for these effects, since we used an enriched serum endowed with a “whole metabolites signal”. In any case, even if from a mechanistic point of view, it might be interesting to decipher the contribution of each compound separately, from a nutritional point of view, it makes sense to consider that the observed biological activity likely results from the synergistic impact of the different compounds that are found in the extract. The detection and quantification of saffron’s compounds after saffron extract ingestion in biological samples requires appropriate analytical methods. Although we can find some precedence in the literature for the quantification of the free crocetin form [87,88], no analytical methods have been described for the detection of conjugated metabolites (such as glucuronides, sulfates, methylates, etc.), safranal, picrocrocin, and derivatives in human blood. Work on developing this type of analytical method is therefore necessary before being able to identify which native and/or metabolized compounds are really involved in the observed effects. Such methods may be required for further pharmaceutical development and optimized therapeutic strategies. One may also question the cellular models. For chondrocytes, we used primary cells, and we validated their sensitivity to inflammatory stress. This 2D cell culture is a well-acknowledged physiological model for studying articular conditions; however, investigations could be confirmed in 3D models, even if they also have their drawbacks, since cell exposure to bioactives may be more random. In addition, for enterocytes, we used a human cell line rather than primary cells, and enterocytes are not the only cell type that is involved in the intestinal barrier homeostasis. Caco-2 cells have been widely reported as a relevant model for investigations of intestinal tissues; however, further investigations on other cell types such as paneth cells, goblet cells, and endocrine cells may contribute to fully evaluating the potential health benefits of saffron extract on the intestinal epithelium.

In conclusion, we provided here new findings on the efficacy and mechanism of circulating saffron compounds and/or metabolites on chondrocyte and enterocyte cells that were submitted to inflammatory stress. These promising results may lead to efficient development and safe plant-based strategies to help patients suffering from chronic inflammatory diseases. Saffron extract could be used in primary prevention in patients with a high risk of developing chronic inflammatory diseases but also as secondary prevention, in association with conventional treatments, in order to reduce the prevalence of these diseases.

## 5. Patents

The human ex vivo methodology used in this study has been registered as a written invention disclosure by the French National Institute for Agronomic, Food and Environment Research (INRAE) (DIRV#18-0058). Clinic’n’Cell^®^ has been registered as a trademark. Safr’Inside^TM^ used in this study is a patented saffron extract (WO2018020013). 

## Data Availability

The data presented in this study are available on request from the corresponding author. The data are not publicly available due to ethical restrictions.
